# Gray matter alterations and their atlas-based spatial associations with neurotransmitter distributions in isolated REM sleep behavior disorder

**DOI:** 10.1007/s11325-026-03845-2

**Published:** 2026-10-29

**Authors:** Jung-Ick Byun, Huijin Song, Jung Hwan Shin, Han-Joon Kim, Won Chul Shin, Chul-Ho Sohn, Ki-Young Jung

**Affiliations:** 1 Department of Neurology, Kyung Hee University Hospital at Gangdong, Kyung Hee University College of Medicine, Seoul, Korea; 2 Departments of Radiology, Seoul National University Hospital, Seoul National University College of Medicine, 101 Daehak-Ro, Jongno-Gu, Seoul, 110-744 Korea; 3 Department of Neurology, Seoul National University Hospital, Seoul National University College of Medicine, 101 Daehak-Ro, Jongno-Gu, Seoul, 110-744 Korea

**Keywords:** REM sleep behavior disorder, Phenoconversion, Magnetic resonance imaging, Voxel-based morphometry, Neurotransmitter

## Abstract

**Purpose:**

Multiple neurotransmitter systems are implicated in the pathophysiology of isolated rapid eye movement sleep behavior disorder (iRBD) and its progression to α-synucleinopathy. This study aimed to identify baseline gray matter (GM) alterations in patients with iRBD compared with healthy controls and to examine their spatial correspondence with known neurotransmitter distributions. We further characterized GM patterns associated with future phenoconversion and their neurochemical context.

**Methods:**

A total of 109 patients with iRBD who completed at least one follow-up evaluation and 24 age- and sex-matched healthy controls underwent structural magnetic resonance imaging. Gray matter volume (GMV) alterations were assessed using the CAT12 toolbox. Spatial correlations between group-level GMV difference maps and neurotransmitter atlases were evaluated using the JuSpace toolbox.

**Results:**

At baseline, patients with iRBD showed reduced GMV in the right cerebellar cortex and vermis, along with increased GMV in the left putamen and pallidum. These GMV alterations demonstrated significant spatial correspondence with dopaminergic receptor and transporter maps, including dopamine D2 receptors and the dopamine transporter (DAT). Among patients with iRBD, those who subsequently phenoconverted exhibited lower baseline GMV in the right medial orbital gyrus compared with non-converters. This pattern showed significant spatial correlations with serotonergic (5-HT2A), glutamatergic (mGluR5), GABAergic (GABAa), and dopaminergic (FDOPA) neurotransmitter maps.

**Conclusion:**

Patients with iRBD exhibit distinct patterns of GMV alteration that spatially align with dopaminergic neurotransmitter distribution maps, while phenoconversion is associated with GM changes corresponding to multiple neurotransmitter systems, although the phenoconversion findings are exploratory.

**Supplementary Information:**

The online version contains supplementary material available at https://doi.org/10.1007/s11325-026-03845-2.

## Introduction

Most patients with isolated rapid eye movement (REM) sleep behavior disorder (iRBD) eventually develop neurodegenerative diseases associated with α-synuclein pathology, and iRBD is therefore widely regarded as a prodromal stage of synucleinopathy [1]. Accumulating evidence suggests that iRBD shares common neurodegenerative processes with established α-synucleinopathies, including Parkinson’s disease (PD) and dementia with Lewy bodies [2].

In PD, motor symptoms are primarily linked to degeneration of the nigrostriatal dopaminergic system, whereas non-motor symptoms are associated with broader involvement of cholinergic, serotonergic, glutamatergic, and noradrenergic neurotransmitter systems [3]. As a prodromal form of α-synucleinopathy, dopaminergic loss has been demonstrated in patients with RBD [4]. Furthermore, multimodal imaging studies have shown that RBD is accompanied by alterations across multiple neurotransmitter systems, including cholinergic, noradrenergic, and dopaminergic circuits [5].

Structural neuroimaging studies have reported gray matter (GM) alterations in patients with idiopathic RBD, involving both subcortical structures like the caudate nucleus and various prefrontal and temporal cortical areas [6]. These GM changes are thought to reflect regional neuronal vulnerability that may be associated with specific neurotransmitter systems [7]. Supporting this notion, recent evidence indicates that regional variability in neurotransmitter distributions is intrinsically linked to patterns of structural brain modification [5]. Accordingly, assessing the spatial correspondence between GM alterations and neurotransmitter maps may provide insights into the neurobiological substrates underlying RBD and its progression toward overt neurodegeneration.

In this study, we aimed to evaluate baseline GM alterations in patients with iRBD compared with healthy controls and to examine their spatial correspondence with established neurotransmitter distributions. In addition, we investigated GM patterns associated with phenoconversion and characterized their corresponding neurochemical profiles. To this end, we employed the JuSpace toolbox to assess spatial correlations between GM difference maps and PET/SPECT-derived neurotransmitter atlases [8], a methodology that has been validated in studies of PD [9], as well as other neurodegenerative disorders, including frontotemporal dementia and Alzheimer’s disease [10].

## Materials and methods

### Study participants

Patients with video-polysomnography–confirmed iRBD who underwent T1-weighted magnetic resonance imaging (MRI) at Seoul National University Hospital were consecutively recruited. To evaluate the phenoconversion, we included only patients who completed at least one longitudinal follow-up evaluation. The diagnosis of iRBD was established according to the International Classification of Sleep Disorders, Third Edition (ICSD-3) criteria [11].

Participants were excluded if they met any of the following criteria: (1) a history of other pre-existing neurodegenerative diseases, (2) moderate-to-severe obstructive sleep apnea (defined as an apnea-hypopnea index ≥ 15/hr), or (3) significant structural brain lesions identified on neuroimaging. The control group consisted of 24 age- and sex-matched healthy volunteers without sleep-related, neurological (including dementia or parkinsonism), or psychiatric disorders. Controls were examined by a neurologist and screened by interview for RBD symptoms, parkinsonism, and cognitive impairment; none had a MoCA-K score below 25. Controls did not undergo video-polysomnography.

The study protocol was approved by the Institutional Review Board of Seoul National University Hospital (IRB No. 1702-150-835), and written informed consent was obtained from all participants prior to enrollment.

### Clinical evaluation

The severity and frequency of RBD symptoms were assessed using the REM Sleep Behavior Disorder Questionnaire–Korean version (RBDQ-KR), a validated 13-item instrument derived from the original Hong Kong version [12]. Global cognitive function was measured using the Montreal Cognitive Assessment (MoCA) [13]. Additionally, depressive symptoms were quantified using the Korean version of the Geriatric Depression Scale (GDS-K) [14].

### Follow-up evaluation

The follow-up protocol has been described in detail previously [15]. Briefly, two neurologists (K.Y.J., H.J.K.) examined each patient every 6 to 12 months, blinded to the imaging analyses. Parkinson’s disease, dementia with Lewy bodies (DLB), and multiple system atrophy (MSA) were diagnosed according to the Movement Disorder Society [16], DLB Consortium [17], and second consensus (Gilman) criteria [18], respectively. Patients who developed one of these disorders were classified as phenoconverters, and those who had not by their last follow-up visit as non-converters (censored and still at risk).

### Image acquisition and preprocessing

Structural MRI data were acquired on a 3.0-Tesla Siemens Biograph mMR scanner (Siemens Healthcare Sector, Germany) equipped with a 16-channel head coil. T1-weighted images were obtained using a three-dimensional magnetization-prepared rapid gradient-echo (MPRAGE) sequence with the following parameters: repetition time (TR) = 1670 ms, echo time (TE) = 1.89 ms, flip angle = 9°, field of view (FOV) = 250 mm, acquisition matrix = 256 × 256, voxel size = 1.0 × 1.0 × 1.0 mm³, and 208 sagittal slices. All images were visually inspected by an experienced neuroradiologist to ensure image quality and to exclude incidental structural abnormalities or artifacts.

Image preprocessing was performed using the Computational Anatomy Toolbox (CAT12) implemented in Statistical Parametric Mapping software (SPM12; Wellcome Centre for Human Neuroimaging) running on MATLAB R2024b (MathWorks, Natick, MA) [19]. T1-weighted images were segmented into gray matter, white matter, and cerebrospinal fluid components using default CAT12 settings.

### GMV analysis

Voxel-based morphometry (VBM) was performed on segmented gray matter (GM) images using the CAT12 toolbox. Individual GM maps were spatially normalized to the Montreal Neurological Institute (MNI) space using affine and nonlinear registration and modulated with Jacobian determinants to preserve absolute GM volume at each voxel. The modulated GM images were smoothed with an isotropic Gaussian kernel of 8-mm full width at half maximum (FWHM) to improve the signal-to-noise ratio and account for inter-individual anatomical variability.

### Spatial correlation with neurotransmitter density maps

Spatial correlations between gray matter morphological alterations and neurotransmitter distributions were assessed using the JuSpace toolbox (version 2.0). Unthresholded voxel-wise t-statistic maps derived from group-level GMV comparisons (iRBD vs. HCs, and phenoconverters vs. non-converters) were used as input for the spatial correlation analyses.

These maps were correlated with PET/SPECT-derived neurotransmitter atlases encompassing serotonergic (5-HT1A, 5-HT1B, and 5-HT2A receptors, serotonin transporter [SERT]), dopaminergic (dopamine D1 and D2 receptors, dopamine transporter [DAT], and [¹⁸F]-FDOPA), GABAergic (GABAa), opioid (µ-opioid receptor), cannabinoid (CB1), noradrenergic (norepinephrine transporter [NAT]), cholinergic (vesicular acetylcholine transporter [VAChT]), and glutamatergic (metabotropic glutamate receptor 5 [mGluR5]) systems.

### Statistical analysis

Demographic and clinical variables were compared between groups using independent-sample t tests or Mann–Whitney U tests for continuous variables, and chi-square tests for categorical variables, as appropriate. Continuous data are reported as mean ± standard deviation, as appropriate. All clinical statistical analyses were performed using SPSS software (version 22.0; IBM Corp., Armonk, NY, USA).

Group differences in gray matter volume (GMV) were examined using a general linear model (GLM) implemented in SPM12. Separate models were constructed to compare (1) patients with iRBD versus healthy controls and (2) phenoconverters versus non-converters. To control for potential confounding effects, total intracranial volume (TIV) was included as a nuisance covariate, and age, sex, and years of education were entered as covariates in all models. Statistical significance was defined at a voxel-level threshold of *p* < 0.001 (uncorrected), followed by cluster-level family-wise error (FWE) correction at *p* < 0.05. As an exploratory time-to-event analysis, we fitted a Cox proportional-hazards model with the time from MRI to phenoconversion (or last follow-up) as the outcome and the mean gray matter volume of the orbitofrontal cluster as the predictor, adjusted for age, sex, education, and TIV.

For spatial correlation analyses, Spearman’s rank correlation coefficients were calculated between unthresholded voxel-wise t-statistic maps and each neurotransmitter atlas using the JuSpace toolbox (version 2.0). Spatial correlations were computed using the unthresholded t-maps of the iRBD > control and non-converter > converter contrasts, such that positive t values indicate greater gray matter volume in patients with iRBD and in non-converters, respectively. Correlation coefficients were Fisher’s z-transformed to normalize their distribution. The statistical significance was assessed using 10,000 exact spatial permutations, in which the null distribution is generated from surrogate maps that preserve the spatial autocorrelation structure of the maps. To control for multiple comparisons across neurotransmitter maps, false discovery rate (FDR) correction was applied with a significance threshold of *p* < 0.05.

## Results

### Patient characteristics

The mean age of the patients with iRBD was 69 years, with 56 participants (51.4%) being male, which was comparable to the HC group. The mean RBDQ-KR score was 48.7 ± 18.7. Patients with iRBD showed higher scores on the Geriatric Depression Scale–Korean version (GDS-K; 9.7 ± 7.6) compared with controls. The mean MoCA-K score in the iRBD group was 26.1 ± 2.7, which was lower than that of the control group (Table 1).

**Table 1:** Demographic and clinical characteristics of patients with isolated REM sleep behavior disorder (iRBD) and healthy controls

	Controls	iRBD	*p*-value
	*n* = 24	*n* = 109	
Age (years)	67.2 ± 3.6	68.8 ± 5.6	0.173
Sex, male (%)	13 (54.2)	56 (51.4)	0.804
BMI (kg/m^2^)	22.8 ± 2.4	23.7 ± 2.8	0.125
Education (years)	14.1 ± 2.6	12.7 ± 3.9	0.097
RBDQ-KR	3.9 ± 2.6	48.7 ± 18.7	< 0.001
MoCA-K	27.4 ± 1.2	26.1 ± 2.7	0.020
GDS-K	2.9 ± 3.2	9.7 ± 7.6	< 0.001
TIV	1497.6 ± 123.2	1482.4 ± 127.9	0.590

Abbreviation: iRBD, isolated rapid eye movement sleep behavior disorder; BMI, body mass index; RBDQ-KR, REM sleep behavior disorder questionnaire–Korean version; MoCA-K, Korean version of the Montreal Cognitive Assessment; GDS-K, Korean version of the Geriatric Depression Scale; TIV, total intracranial volume

Participants were followed for a mean duration of 4.5 ± 1.2 years. During this period, 16 patients (14.7%) phenoconverted to a neurodegenerative disorder, including Parkinson’s disease (*n* = 12), multiple system atrophy (*n* = 3), and dementia with Lewy bodies (*n* = 1). The mean interval from baseline to conversion was 3.9 ± 2.0 years. Phenoconverters tended to have a lower body mass index (BMI) and higher GDS-K scores at baseline compared with non-converters. (Supplementary Table 1).

### Gray matter volume alterations

**Fig. 1:**
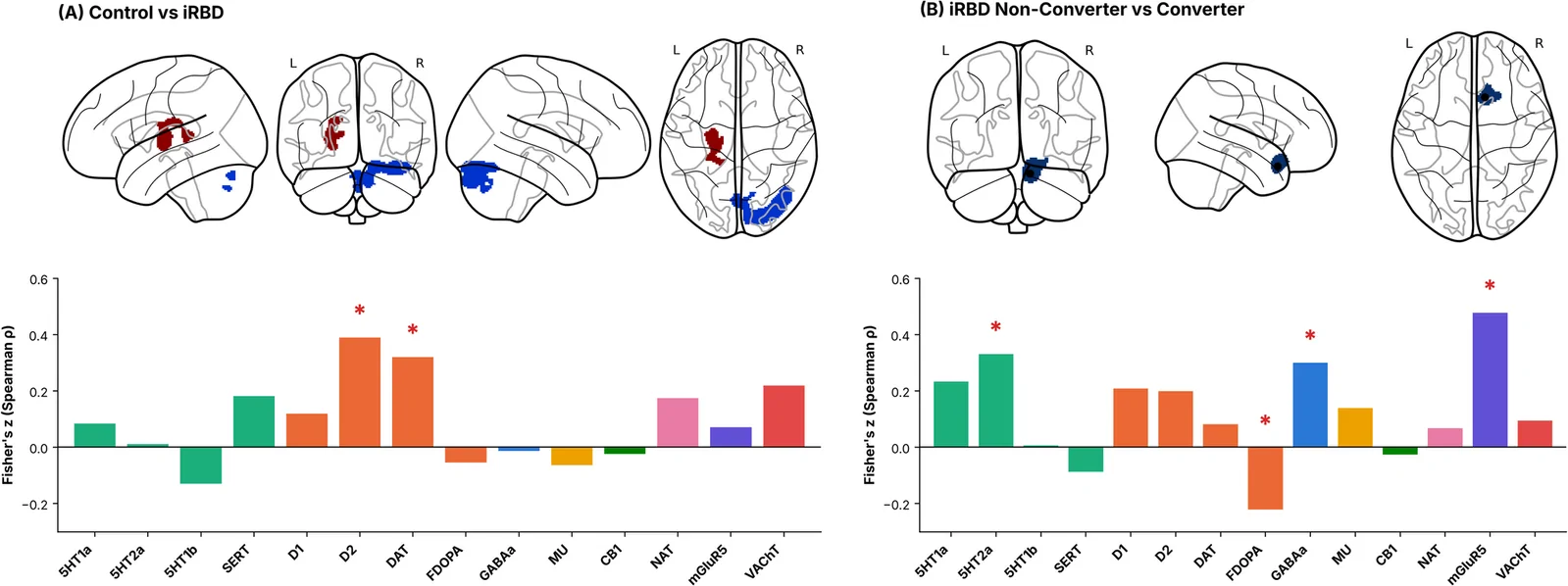
Spatial correlation analysis between voxel-wise gray matter volume (GMV) alterations and neurotransmitter distribution maps. (**A**) iRBD > control, (**B**) Non-converter > converter. Upper panels show clusters of significant GMV differences (voxel-level *p* < 0.001, cluster-level FWE-corrected *p* < 0.05) rendered on glass-brain templates. Red indicates increased and blue decreased GMV in patients with iRBD relative to controls (**A**), and in phenoconverters relative to non-converters (**B**). Lower panels show Fisher’s z–transformed partial Spearman correlations between the unthresholded t-map of each contrast and normative neurotransmitter distribution maps, controlling for the tissue-probability map. The corresponding Spearman’s ρ values are reported in Table 3. Positive values in (**A**) indicate greater GMV in iRBD in regions of higher tracer density, and in (**B**) greater GMV in non-converters (i.e., lower GMV in phenoconverters). Statistical significance was assessed using 10,000 exact spatial permutations that preserve the spatial autocorrelation structure of the maps. Asterisks indicate correlations surviving false discovery rate (FDR) correction (*p* < 0.05). Abbreviations: iRBD, isolated REM sleep behavior disorder; GMV, gray matter volume; FWE, family-wise error; FDR, false discovery rate; 5HT1a, 5HT1b, 5HT2a, serotonin receptor subtypes; SERT, serotonin transporter; D1, D2, dopamine receptors; DAT, dopamine transporter; FDOPA, fluorodopa; GABAa, γ-aminobutyric acid type A receptor; MU, µ-opioid receptor; CB1, cannabinoid receptor type 1; NAT, noradrenaline transporter; mGluR5, metabotropic glutamate receptor 5; VAChT, vesicular acetylcholine transporter

VBM analysis identified distinct patterns of structural brain changes in patients with iRBD. Compared with healthy controls, patients with iRBD exhibited significant reductions in gray matter volume (GMV) in the cerebellar exterior and vermis, along with increased GMV in the left pallidum and putamen. Within the iRBD cohort, patients who subsequently phenoconverted showed significantly lower baseline GMV in the right medial orbital gyrus compared with non-converters. (Table 2; Fig. 1) In an exploratory Cox proportional-hazards analysis, lower baseline orbitofrontal gray matter volume was associated with a higher hazard of phenoconversion (hazard ratio = 2.9 per 1-SD decrease; 95% CI 1.6–5.2; *p* < 0.001), which persisted after adjustment for age, sex, education, and TIV (HR ≈ 3.1) and for GDS-K (HR ≈ 2.7).

**Table 2:** Brain regions showing significant gray matter volume differences between groups

Cluster	k	Peak cluster *p*	T		Z		Peak region	MNI coordinates		
	(voxels)	(FWE-corrected)						x	y	z
Controls > iRBD	2686	< 0.0001	5.11		4.86	Cbll vermis, exterior		20	-90	-21
Controls < iRBD	1049	0.019	4.39		4.22	Lt. putamen/pallidum		-22	-12	2
Non-converters> Converters	870	0.048	4.63		4.40	Rt. Medial orbital gyrus		10	18	-24

Abbreviation: GMV, gray matter volume; MNI, Montreal Neurological Institute; FWE, family-wise error

### Atlas-based spatial associations of GMV alterations with neurotransmitter distributions

Spatial correlation analyses revealed significant correlations between GMV alterations in patients with iRBD and dopaminergic neurotransmitter maps, including the dopamine D2 receptor (ρ = 0.37, FDR-corrected *p* = 0.001) and the DAT (ρ = 0.31, FDR-corrected *p* = 0.004). The spatial pattern of GMV differences between phenoconverters and non-converters demonstrated significant correlations with serotonergic (5-HT2a; ρ = 0.32, FDR-corrected *p* = 0.003), glutamatergic (mGluR5; ρ = 0.45, FDR-corrected *p* = 0.004), and GABAergic (GABAa; ρ = 0.29, FDR-corrected *p* = 0.005) neurotransmitter systems. A weaker association was observed for [^18^F]-FDOPA (ρ = −0.22, FDR-corrected *p* = 0.046), whereas the dopamine D1 and D2 receptor maps showed nominal associations that did not survive FDR correction (ρ = 0.21 and 0.20; FDR-corrected *p* = 0.056 and 0.060, respectively). (Table 3; Fig. 1)

**Table 3:** Spatial correlations between group-level gray matter volume changes and neurotransmitter distribution maps

Neurotransmitter	iRBD > Control		Non-converter > Converter	
	Spearman’s rho	FDR-corrected *p*-value	Spearman’s rho	FDR-corrected *p*-value
5HT1a	0.086	0.8424	0.231	0.1104
5HT2a	0.013	0.8867	0.321	0.0028
5HT1b	−0.130	0.3912	0.009	0.9226
SERT	0.182	0.1702	−0.089	0.5196
D1	0.121	0.3912	0.208	0.0557
D2	0.373	0.0014	0.199	0.0602
DAT	0.312	0.0042	0.083	0.5196
FDOPA	−0.056	0.8424	−0.219	0.0458
GABAa	−0.015	0.8867	0.293	0.0051
Mu	−0.065	0.8867	0.140	0.6267
CB1	−0.025	0.8867	−0.028	0.9012
NAT	0.175	0.1702	0.069	0.5781
mGluR5	0.073	0.8787	0.446	0.0042
VAChT	0.218	0.0709	0.096	0.5069

Abbreviations: 5-HT, serotonin; SERT, serotonin transporter; DAT, dopamine transporter; FDOPA, 6-[¹⁸F]-fluoro-L-DOPA; GABAa, gamma-aminobutyric acid type A receptor; CB1, cannabinoid receptor type 1; NAT, norepinephrine transporter; mGluR5, metabotropic glutamate receptor 5; VAChT, vesicular acetylcholine transporter; FDR, false discovery rate

## Discussion

This study identified distinct patterns of GMV alterations associated with iRBD and its future phenoconversion, and examined the spatial correspondence between these structural changes and established neurotransmitter distributions. Compared with healthy controls, patients with iRBD exhibited reduced GMV in the cerebellar exterior and vermis, along with increased GMV in the left putamen and pallidum, which showed spatial alignment with dopaminergic receptor and transporter distributions. In addition, patients who subsequently phenoconverted demonstrated reduced baseline GMV in the right medial orbital gyrus, a region whose spatial pattern corresponded predominantly to serotonergic (5-HT2A), glutamatergic (mGluR5), and GABAergic (GABAa) receptor distributions.

Structural alterations involving the cerebellum have been consistently reported in iRBD. Previous neuroimaging studies have described both cerebellar GMV increases [6] and decreases [20] in patients with iRBD. In the present study, we observed GMV reduction predominantly involving the right posterior cerebellum, which anatomically overlaps with the region corresponding to lobule VIIB reported in the previous study [20]. Our results further support a potential role of the cerebellum in the pathogenesis of iRBD. Increased GMV in the putamen has also been reported in iRBD [21], although findings across studies have been heterogeneous [22]. These regional GMV increases may reflect adaptive or compensatory change [23], but alternative explanations—including tissue-segmentation effects related to the signal properties of the basal ganglia, iron-related influences on T1 contrast, glial or inflammatory changes, vascular or metabolic factors, and registration bias—cannot be excluded, and a compensatory mechanism cannot be confirmed without longitudinal, diffusion, susceptibility, or molecular imaging.

The spatial pattern of GMV alterations observed in iRBD showed significant correspondence with dopaminergic maps, particularly D2 receptor and DAT atlases, consistent with prior imaging studies [4]. Given the high density of D2 receptors and the dopamine transporter in the striatum and the striatal location of one of the clusters, this correspondence should be interpreted as an atlas-based spatial association rather than evidence of a specific dopaminergic mechanism. Cortical thickness alterations in iRBD have previously been shown to spatially align with DAT density maps [24], and presynaptic dopaminergic imaging studies have demonstrated substantial nigrostriatal dysfunction in iRBD, with more pronounced tracer reductions in patients exhibiting subtle motor features [25, 26]. Meta-analytic evidence further supports reduced nigrostriatal dopaminergic function in idiopathic RBD, particularly in the putamen [27].

Within the iRBD cohort, reduced baseline GMV in the right medial orbital gyrus was associated with subsequent phenoconversion. This finding is in line with previous reports identifying frontal cortical atrophy, including the inferior frontal gyrus, as a structural feature associated with progression to α-synucleinopathies [28]. The medial orbital region is also considered part of an olfactory-related cortical network [29], dysfunction of which represents a hallmark prodromal feature of synucleinopathies [30]. Consistent with this notion, olfactory impairment in iRBD has been associated with an increased risk of phenoconversion over relatively short follow-up periods [31, 32].

Among the neurotransmitter systems examined, mGluR5 showed the largest effect size (ρ = 0.45), whereas the 5-HT2A atlas showed the lowest FDR-corrected p value. This finding aligns with evidence that elevated 5-HT2A receptor binding in the frontal cortex of patients with Parkinson’s disease is associated with increased α-synuclein burden [33]. In the context of RBD, serotonergic pathways play an important role in the regulation of REM sleep without atonia [34]. Although pharmacological modulation of 5-HT2A receptors has not resulted in consistent symptomatic improvement in RBD [35], the present findings suggest that serotonergic receptor architecture may characterize cortical regions that are structurally vulnerable during phenoconversion and may provide neurochemical context for the underlying neurodegenerative trajectory.

Glutamatergic signaling, as captured by the mGluR5 receptor map, also showed a robust spatial correspondence with GMV alterations associated with phenoconversion. mGluR5 is a G protein–coupled receptor that modulates N-methyl-D-aspartate (NMDA) receptor activity and has been implicated in neurodegenerative cascades. Experimental studies have demonstrated that mGluR5 activation can exacerbate excitotoxic neuronal injury [36], whereas pharmacological inhibition of mGluR5 attenuates axonal degeneration in animal models of PD [37]. In line with these findings, a recent PET study reported elevated mGluR5 binding in patients with PD and comorbid RBD, which correlated with the severity of REM sleep without atonia [38].

The spatial correspondence with GABAA distributions may provide an indirect neurochemical context for the structural changes in iRBD, although GABAergic function was not measured in this study. Animal models have demonstrated that impaired GABAa-mediated inhibition is associated with RBD-like behaviors [39]. Moreover, studies in early-stage Parkinson’s disease have reported divergent alterations in basal ganglia GABA levels depending on the presence of RBD, despite comparable nigral iron and neuromelanin burdens [40]. Together, these findings support a role for GABAergic circuitry in shaping vulnerability patterns observed in prodromal synucleinopathies.

The negative correlation with FDOPA, although weak (ρ = −0.22, FDR-corrected *p* = 0.046), may suggest that brain regions with lower dopaminergic synthesis capacity are more susceptible to GMV reduction associated with phenoconversion in iRBD. Previous imaging studies have reported reduced AADC activity in the putamen [5, 41] and thalamus [41] in patients with iRBD compared with healthy controls. Moreover, reduced AADC activity has been linked to increased microglial activation, which may contribute to structural brain changes [41]. Given the weak and borderline correlation, however, this interpretation remains speculative.

Several limitations of this study should be acknowledged. First, both the healthy control group (*n* = 24) and the subset of patients who phenoconverted (*n* = 16) were relatively small. Although statistical significance for voxel-based morphometry analyses was determined using a cluster-level FWE correction following an initial voxel-level threshold, the limited sample size may still affect the stability and generalizability of the findings. Second, prodromal assessments such as MDS-UPDRS-III and olfactory testing were not performed in all patients and did not differ between groups in those assessed. We therefore cannot exclude that the orbitofrontal finding partly identifies patients with more advanced prodromal disease at baseline. Patients with iRBD had poorer cognition and higher depressive-symptom scores than controls; these differences may partly contribute to the structural findings. The phenoconversion-related findings did not survive adjustment for GDS-K; because depressive symptoms are themselves a prodromal feature of synucleinopathy, this may represent partial over-control, and these findings should therefore be regarded as exploratory. Controls did not undergo video-polysomnography, so unrecognized REM sleep without atonia cannot be excluded. Third, JuSpace analyses rely on neurotransmitter atlases derived from healthy individuals and therefore provide only indirect information regarding neurochemical alterations in patients. Consequently, causal inferences regarding specific neurotransmitter systems cannot be drawn from the present findings alone and should be addressed in future studies using subject-level molecular imaging. Finally, because spatial correlation analyses were performed using group-level t-statistic maps, we were unable to assess direct associations between individual-level structural brain measures and specific clinical variables. Moreover, whole-brain VBM with 8-mm smoothing has limited sensitivity for the small pontine and medullary structures central to REM atonia; the absence of brainstem findings therefore does not exclude brainstem involvement, and our results should not be regarded as a complete account of iRBD pathophysiology. Because the outcomes were heterogeneous (Parkinson’s disease, MSA, and DLB), these findings reflect prodromal synucleinopathy in general and cannot be attributed to a single disorder. Therefore this study does not provide an individual-level prediction model, internal or external validation, and the results represent group-level differences rather than a validated prognostic biomarker.

## Conclusion

GMV alterations in patients with iRBD demonstrate significant spatial alignment with the distribution of dopaminergic receptors and transporters. In addition, structural atrophy associated with future phenoconversion shows spatial correspondence with serotonergic (5-HT2A), glutamatergic (mGluR5), and GABAergic (GABAA) distribution maps. Although based on indirect spatial correlations, these findings provide a neurochemical context for regional brain vulnerability in iRBD; they represent group-level differences rather than a validated prognostic biomarker.

## Supplementary Information

Below is the link to the electronic supplementary material.

Supplementary Material 1 (DOCX 15.9 KB)

## Data Availability

All relevant data are provided within the manuscript.
