# Supplementary material for: Gray matter alterations and their atlas-based spatial associations with neurotransmitter distributions in isolated REM sleep behavior disorder
Source: Sleep Breath. 2026 Oct 29;30(6):300. doi: 10.1007/s11325-026-03845-2 (PMC13723558; doi:10.1007/s11325-026-03845-2)
Supplement: Supplementary file 1 [file 11325_2026_3845_MOESM1_ESM.docx]

Supplementary Table 1. Demographic and clinical characteristics of iRBD patients according to phenoconversion status

|  | Non-converter | Converter | p-value |
| --- | --- | --- | --- |
|  | n=93 | n=16 |  |
| Age (years) | 68.5±5.7 | 70.6±4.8 | 0.132 |
| Sex, male (%) | 50 (53.8) | 6 (37.5) | 0.229 |
| BMI (kg/m^2^) | 23.9±2.8 | 22.7±2.1 | 0.058 |
| Education (years) | 13.0±3.8 | 11.4±4.0 | 0.148 |
| RBDQ-KR | 48.8±19.3 | 48.1±15.0 | 0.861 |
| MoCA-K | 26.1±2.6 | 26.0±3.1 | 0.933 |
| GDS-K | 9.1±7.5 | 13.9±7.6 | 0.033 |
| TIV | 1489.6±123.8 | 1440.6±147.0 | 0.223 |

Abbreviation: iRBD, isolated rapid eye movement sleep behavior disorder; BMI, body mass index; RBDQ-KR, REM sleep behavior disorder questionnaire–Korean version; MoCA-K, Korean version of the Montreal Cognitive Assessment; GDS-K, Korean version of the Geriatric Depression Scale; TIV, total intracranial volume.
